# The role of life skills in developing an authentic leadership attitude in public health students: a multicenter cross-sectional study in Poland

**DOI:** 10.1186/s12889-022-13907-1

**Published:** 2022-08-04

**Authors:** Mariusz Jaworski, Mariusz Panczyk, Ilona Cieślak, Agata Baranowska, Katarzyna Brukało, Jolanta Grzebieluch, Magdalena Kwaśniewska, Monika Urbaniak, Marzena Zarzeczna-Baran, Aleksandra Zyska, Joanna Gotlib

**Affiliations:** 1 Department of Education and Research in Health Sciences, Faculty of Health Sciences, Medical University of Warsaw, 14/16 Litewska Street, 00-581 Warsaw, Poland; 2 Department of Health Promotion, Faculty of Physical Culture and Health Promotion, University of Szczecin, Szczecin, Poland; 3 Department of Health Policy, Faculty of Public Health, Medical University of Silesia in Katowice, Bytom, Poland; 4 Department of Organization and Management, Faculty of Health Sciences, Wroclaw Medical University, Wroclaw, Poland; 5 Department of Preventive Medicine, Faculty of Health Sciences, Medical University of Lodz, Lodz, Poland; 6 Department of Medical and Pharmaceutical Law, Faculty of Health Sciences, Poznan University of Medical Sciences, Poznan, Poland; 7 Center of Competence Development, Integrated Care and e-Health, Medical University of Gdansk, Gdansk, Poland; 8 Academy of Applied Medical and Social Sciences, Elbląg, Poland; 9 Institute of Medical Science, Opole University, Opole, Poland

**Keywords:** Life skills, Leadership competencies, Decision-making skills, Public health, Cross-sectional study

## Abstract

**Background:**

Decision-making skills are considered crucial life skills that condition proper social functioning within groups (i.e., support authentic leadership skills and increasing one’s chances of success and wellbeing in life). Nonetheless, the number of scientific papers addressing the role of life skills in developing authentic leadership skills in public health students is limited. The aim of the present study was to develop a theoretical model to determine the role of selected life skills in developing authentic leadership skills in public health students.

**Methods:**

The study was conducted from January 16 through February 28, 2018. In total, 653 students undertaking in-service training in Master’s degree programs qualified for the study, and complete data sets were obtained from 329 students (response rate 50.38%). The data were collected by means of a paper questionnaire. Four research tools were used in the study: The Authentic Leadership Self-Assessment Questionnaire, The Moral Foundations Questionnaire, The General Self-Efficacy Scale, and The Youth Leadership Life Skills Development Scale.

**Results:**

Two subgroups were identified among the public health students in the study: 1) the extra life skills training group (*N* = 113) and 2) the no extra life skills training group (*N* = 216). Both groups of study participants did not differ significantly in terms of age (M (SD): 25.0 (3.89) vs. 25.0 (3.66); t = 0.068, *P* = 0.946). On the other hand, clear differences were observed in the case of the respondents’ participation in voluntary service. The respondents from the extra life skills training group declared participation in voluntary activities less frequently than the respondents from the second identified group (48.7 vs. 31.9%).

**Conclusions:**

A verified theoretical model showed that course aimed at strengthening authentic leadership competences should be modular, should focus on self-improvement and critical reflection, and should be spread over time to enable and encourage each participant to grow and flourish at their own pace.

## Introduction

The European List of Core Competences for the Public Health (PH) Professional developed by The Association of Schools of Public Health in the European Region identifies effective decision making as a crucial skill for public health specialists [1]. Effective healthcare decision making involves the use of decision-making skills (DMS), i.e., the ability to make the best possible choice to achieve the best possible result [2]. The key role of these skills in public health was emphasized by, among others, Dobbins et al. [3] and McCarthy et al. [4]. Public health professionals shape health policies, are responsible for implementing health education not only in the context of the individual patient but also in the context of entire communities, and make management decisions based on scientific evidence that affect health systems as a whole [5]. It is thought that a lack of decision-making skills in the healthcare setting may result in mistakes and may pose a risk to patient life or health [4].

Decision-making skills are considered crucial for the condition of life skills (LS) and proper group social functioning (i.e., support authentic leadership skills—ALS) and for increasing one’s chances of success and wellbeing in life [6]. This is due to the assumption that authentic leadership skills build on interpersonal relationships and authentic group functioning [7, 8]. The World Health Organization indicates the need to pay special attention to the development and improvement of leadership in health care, treating it as the basis for effective management and teamwork [9]. According to numerous scientific studies, education in the field of leadership should be included in the health studies programs from the first year of higher education [10]. All reports on undergraduate education in public health, both from the United States [11] and from those presenting data from European Union countries [12], indicate an insufficient implementation level of subjects that shape students’ leadership competences.

In their comprehensive framework, Seevers et al. highlight the importance of life skills in the formation of leadership skills [13]. Defined briefly, life skills are abilities for adaptive and positive behavior that enable humans to deal effectively with the demands and challenges of life [14]. Nonetheless, the number of scientific papers addressing the role of life skills in developing authentic leadership skills in public health students is limited.

Decision-making skills can be learned and developed through training programs and courses. For example, Dobbins et al. [3] demonstrated that proper “face-to-face training and active participation aimed at the development of evidence-informed decision making skills created the greatest impact on associated behaviours, knowledge, and skills”. It is worth noting that to some extent, decision-making skills may depend on personality traits, e.g., self-efficacy [15], something that is particularly noticeable in clinical practice [16]. Self-efficacy is a trait that defines an individual’s belief that they are able to take action towards achieving a set goal [17]. A person’s beliefs regarding how to function in a group may constitute another important factor [13]. The belief in equal effort put by each group member in achieving a shared goal (i.e., fairness) and mutual aid based on empathy (i.e., care) may be of particular importance in this context [7, 8, 17].

Therefore, training programs aimed at developing decision-making skills in public health students should take into account not only the methods of improving these skills, but also their dependence on leadership skills and potential personality predispositions that may modulate the relationship between decision-making skills and authentic leadership skills.

The aim of the present study was to develop a theoretical model to determine the role of selected life skills in developing authentic leadership skills in public health students. We focused on verifying the theoretical model that would identify the impact of individual life skills on the level of authentic leadership skills. Main aim of this study was to investigate the relationship between the various dimensions of life skills and the perception of the level of authentic leadership skills by students. Getting to know these relationships indicates places where space for shaping life skills should be introduced. Increasing the level of individual life skills may contribute to an increase in the level of authentic leadership skills. Due to the fact that in Poland there is no universal leadership course for public health students and each university is free to organize such training, the theoretical model which was developed may be the first step to building an effective leadership training.

### Research question and hypotheses

Bearing the above considerations in mind, we developed a theoretical model to determine the role of selected life skills in developing authentic leadership skills in public health students. Therefore, the following research hypothesis was established Hypothesis 1 (H1): there is a relationship between an individual’s dispositions (beliefs about fairness in group cooperation (F), empathic attitude (C/E), self-efficacy, and decision-making skills) and authentic leadership skills. With reference to H1, the following detailed hypotheses were formulated:

H1a:” The beliefs about fairness in group cooperation (F) and self-efficacy will influence decision-making skills, which will, in turn, condition authentic leadership skills in public health students”.

H1b: “Self-efficacy influences the development of leadership skills in public health students”. This detailed hypothesis was formulated on the basis of a literature review in which a strong relationship was noted between self-efficacy and authentic leadership skills in some medical professions [18, 19]. However, such studies have not been conducted among public health students.

H1c:” Self-efficacy and empathic attitudes (C/E) will have both a direct and indirect impact on the development of leadership skills by affecting decision-making skills”. This hypothesis was developed based on studies emphasizing that, apart from self-efficacy, it is empathy that plays an important role in the development of authentic leadership skills [7, 8, 17].

The last main hypothesis was to verify the assumption that the declared participation in life skills training (LST) would differentiate the developed theoretical model as far as the analyzed variables are concerned. Such a hypothesis would allow potential factors that can be effectively modified to be identified as well as an appropriate intervention to be prepared (Hypothesis 2: There are differences in the theoretical model concerning factors that affect the development of leadership skills between the extra LST group and the no extra LST group).

## Method

### Setting and participants

A nationwide cross-sectional multicentre study was conducted using a total of 329 public health students undertaking postgraduate education as part of their Master’s degree. The study was conducted from January 16 to February 28, 2018.

We invited 31 universities, both public and private, to participate in the study. In 2017, there were a total of 1362 students [20] in Master’s degree programs (Level 7 of the European Qualifications Framework). Eight universities decided to participate in the study. In total, 653 students undertaking in-service training in Master’s degree programs were qualified for the study, and complete data sets were obtained from 329 students (response rate 50.38%). With this sample size and the number of public health students in Master’s degree programs in Poland (*N* = 1362), the error margin was 3.17% (95% confidence level).

### Educational context

In Poland, public health education is conducted in line with the Bologna Process. Higher education programs are divided into: Bachelor’s degree studies (first-cycle studies), Master’s degree studies (second-cycle studies), and doctoral studies (third-cycle studies). These programs are conducted independently of each other, and universities have the ability or organize these cycles [21].

During second-cycle study programs, students need to achieve specific learning outcomes in terms of knowledge, skills, and social competences. Having completed a second-cycle study program, the graduate attains the 7th level of the European Qualifications Framework and obtains the professional title of Master of Public Health [22].

During second-cycle studies, students can choose a major (specialization path) that is interesting to them. Due to the non-regulated nature of study programs at the faculty of public health, each university determines its own curriculum and offered specializations. In Poland, there is a wide range of specialization paths, e.g., health education and social marketing, healthcare analytics, clinical research and health technology assessment, or epidemiology with health promotion elements [23] as well as European public health and lifestyle medicine and management in healthcare [24].

### Measures

#### Four research tools were used in the study

The Authentic Leadership Self-Assessment Questionnaire (ALSAQ) developed by Walumbwa et al. [8] and recommended by Northouse [25] was used to perform the self-assessment. The Polish version of the ALSAQ, validated by Panczyk et al. [19], includes 16 items and allows the global indicator of Authentic Leadership skills and its three components: moral processing, self-awareness, and relational transparency to be measured. The Polish version of the ALSAQ has a good internal consistency (Cronbach’s alpha 0.84) and a test−retest analysis confirmed the stability of the measurement for the subscales and particular items. In our study, we only analyzed the global level of authentic leadership skills, i.e., the sum of the points from the three subscales listed above. We chose not to analyze these subscales separately because they correlate with one another.

The Moral Foundations Questionnaire (MFQ) was developed by Graham et al. [26]. The MFQ measures five universal moral foundations of harm/care, fairness/reciprocity, ingroup/loyalty, authority/respect, and purity/sanctity. The codes provide the basis for the evaluation of one’s behaviour for mortality [26]. We used the Polish version of the MFQ-PL questionnaire, which has good validity and reliability [27]. In order to ensure the validity of the psychological test, the study participants provided answers to all of the questions on the MFQ-PL questionnaire. However, based on the literature review, we only took the results from two subscales, i.e., harm/care and fairness/reciprocity, in account. The harm/care (KODT) subscale refers to empathy and compassion and the principles of not hurting other people and helping those who are weaker and people in need [28]. This subscale was selected for the study because it reflects an empathic attitude, which is a key aspect of authentic leadership [7]. The fairness/reciprocity (KODS) subscale refers to the reciprocity of help given and helping others in contrast to taking advantage of other people and only feigned involvement in action [28]. This subscale was chosen for the study because it is consistent with the general concept of authentic leadership, which involves treating others equally, providing equal opportunities, and acting for the benefit of the group [7].

The General Self-Efficacy Scale (GSES) was originally developed by Matthias Jerusalem and Ralf Schwarzer in 1981 and was designed to assess optimistic self-beliefs and the ability to cope with a variety of difficult situations in life. It is a short 10-item psychometric scale [15]. The scale is one-dimensional and enables a global self-efficacy measurement. We used the Polish version of the GSES, which has good validity and reliability [29].

The Youth Leadership Life Skills Development (YLLSD) Scale was developed by Seevers et al. based on Miller’s concept of leadership life skills development [13]. The YLLSD Scale contains 30 items from seven domains (communication skills, decision-making skills, skills for getting along with others, learning skills, management skills, skills for understanding yourself, and skills for working with groups) that together form a complete picture of leadership skills. The final summated scale of 30 indicators had a Cronbach’s alpha reliability coefficient of .98. In our study, we chose to only analyze decision-making skills, as these skills appear to be crucial for public health specialists [1].

Additionally, the research tool was supplemented with a question concerning declared participation in social skills training. For this purpose, the following yes/no question was asked: Have you participated in training/workshops on non-technical skills (e.g., leadership, communication, social competences, etc.)? Based on the answers to this question, the study group was divided into two subgroups. The first group comprised students who declared that they had participated in at least one life skills training session (extra LST group), while the second group comprised students who declared that they had not participated in such training (no extra LST group).

### Model assumptions

**Fig. 1:**
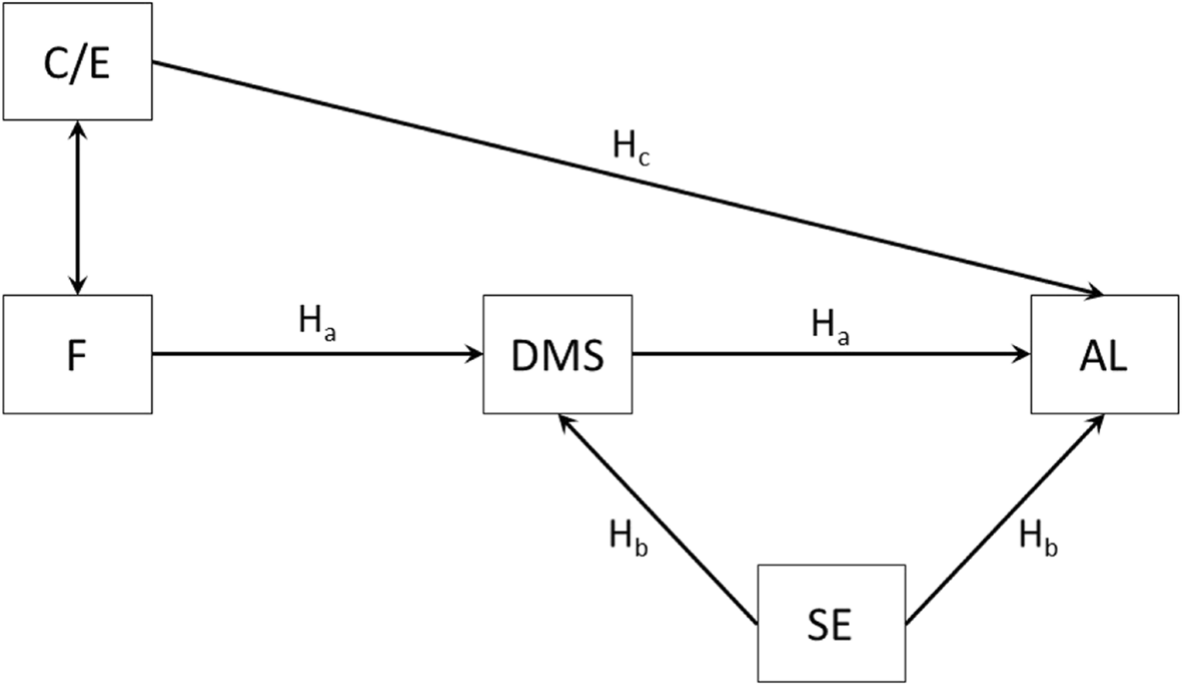
A theoretical model of the relationship between variables. AL—authentic leadership, C/E—care/empathy, DMS—decision-making skills, SE—self-efficacy, F—fairness, H—hypothesis

Based on the formulated hypotheses (H1a–H1c), a theoretical model was developed (Fig. 1) by assuming the impact of self-efficacy (GSES) and fairness in group cooperation (F) on decision-making skills (DMS). Moreover, DMS influence the development of authentic leadership skills (ALS). Empathic attitudes (C/E) are an important addition to this model, as they directly affect ALS and interact with F.

### Data collection

The data were collected by means of a paper questionnaire distributed among a group of students at the end of regular university classes. Trained interviewers limited themselves to stating the aim of the study and informing the participants how to fill in the questionnaire. They were also responsible for collecting the completed questionnaires and securing them prior to sending them to the central unit coordinating the study. ABBYY® FlexiCapture version 9.0 software was used to digitize the paper questionnaire data. Questionnaires with missing data were rejected and were not included in the analysis.

### Data analysis

In order to analyze the variables collected in the study, we used descriptive statistics (mean, standard deviation) and structure coefficients (numbers and frequency). The chi-square independence test and Student’s t test were used to compare the two subgroups (extra life skills training group vs. no extra life skills training group) in terms of the examined characteristics depending on the type of variable (categorical or continuous variables, respectively). The calculations were performed with the use of the STATISTICA package, version 13.3 (Tibco Software Inc., Palo Alto, CA, United States). A 5% level of significance was set.

**Fig. 2:**
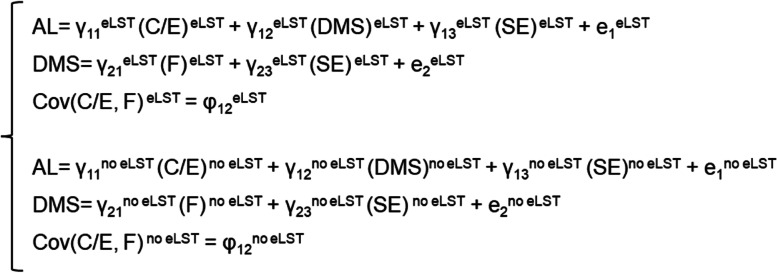
The formal form of the structural equation model. AL—authentic leadership, C/E—care/empathy, DMS—decision-making skills, SE—self-efficacy, F—fairness, eLST—extra life skills training group, no eLST—no extra life skills training group, e – residual, γ – path coefficient, φ – correlation coefficient, Cov – covariant

All analyses were carried out using the structural equation modelling software program Mplus version 7.0 [30]. We used two-group structural equation modelling: extra LST vs. no extra LST groups. The aim of this analysis was to determine whether the relationships between the variables that were assumed theoretically would be confirmed by the collected empirical data. For this purpose, the model parameters (path coefficients, variance, and covariance) were estimated and used to build the theoretical variance-covariance matrix of the variables used in the model (Fig. 2). We verified whether the calculated model parameters differed in the extra LST vs. no extra LST groups. Maximum likelihood estimation with robust standard errors was used to calculate the parameters of the structural model.

The fit of the model was assessed via the following statistics and indices: the chi-square test of model fit (CMIN), normal chi-square (CMIN/DF), the Comparative Fit Index (CFI), the Tucker–Lewis Index (TLI), the Root Mean Square Error of Approximation (RMSEA), and the Standardized Root Mean Square Residual (SRMR). For the evaluation of the model, the chi-square statistics were expected to be nonsignificant. Both the CFI and TLI evaluate the fit of a user-specified solution in relation to a more restricted nested baseline model, in which the covariance among all of the input indicators is fixed to zero or has no relationship among the variables that are posited; in other words, the number of dependent variables is equal to the number of factors. The TLI imposes an additional correction for over-parameterization [31]. The expected values of recommended indices were as follows: χ2 divided by the degrees of freedom (CMIN/DF) ≤ 3.00; RMSEA < 0.080 and SRMR < 0.050; and CFI and TLI > 0.95 [32].

## Results

### Participant characteristics

Two subgroups were identified among the public health students in the study: 1) the extra life skills training group (*N* = 113) and 2) the no extra life skills training group (*N* = 216). Both groups of study participants did not differ significantly in terms of age (M [SD]: 25.0 (3.89) vs. 25.0 (3.66); t = 0.068, *P* = 0.946) or work experience (M (SD): 3.4 (4.88) vs. 3.1 (4.32); t = 0.630, *P* = 0.529). In terms of selected features, the groups differed slightly in terms of the year of study and the frequency of studying at another faculty at the same time. On the other hand, clear differences were observed in the case of the respondents’ participation in voluntary service. The respondents from the extra LST group declared participation in voluntary activities less frequently than the respondents from the no extra LST group (48.7 vs. 31.9%). Table 1 shows a comparison of selected demographic characteristics of the study participants.

**Table 1:** Participant characteristics

	Total (*N* = 329)		Extra LST group (*N* = 113)		No extra LST group (*N* = 216)		χ2	*p*-value*
	N	%	N	%`	N	%		
University								
Poznań University of Medical Sciences	35	10.6	12	10.6	23	10.6	11.276	0.127
Czestochowa University of Technology	32	9.7	8	7.1	24	11.1		
Medical University of Silesia	37	11.2	7	6.2	30	13.9		
Medical University of Gdańsk	21	6.4	5	4.4	16	7.4		
Medical University of Łódź	21	6.4	8	7.1	13	6.0		
Pomeranian Medical University	14	4.3	3	2.7	11	5.1		
Wroclaw Medical University	29	8.8	13	11.5	16	7.4		
Medical University of Warsaw	140	42.6	57	50.4	83	38.4		
**Year of study**								
1	121	36.8	50	44.2	71	32.9	4.130	0.042
2	208	63.2	63	55.8	145	67.1		
**Sex**								
F	278	84.5	96	85.0	182	84.3	0.027	0.868
M	51	15.5	17	15.0	34	15.7		
**Place of residence**								
Village	58	17.6	17	15.0	41	19.0	5.524	0.137
City up to 100,000 inhabitants	70	21.3	22	19.5	48	22.2		
City 100,000–500,000 inhabitants	72	21.9	20	17.7	52	24.1		
City above 500,000 inhabitants	129	39.2	54	47.8	75	34.7		
**Another faculty at the same time**								
No	309	93.9	102	90.3	207	95.8	4.028	0.045
Yes	20	6.1	11	9.7	9	4.2		
**Volunteer work**								
No	205	62.3	58	51.3	147	68.1	8.840	0.003
Yes	124	37.7	55	48.7	69	31.9		
**Professional activity**								
Not working	89	27.1	31	27.4	58	26.9	1.643	0.440
Yes, work not in a profession Related to public health	148	45.0	46	40.7	102	47.2		
Yes, work in a profession related to public health	92	28.0	36	31.9	56	25.9		

* χ ^2^ – chi-squared test

### Variables

All of the analyzed variables were tested for skewness and kurtosis, which showed that they were left-skewed and did not show compliance with the normal distribution. The deviations in terms of compliance with the normal distribution were not very large, as skewness and kurtosis ranged from − 1.5 to + 1.5. All of the variables included in the structural equation model were also analyzed for the presence of data outliers (Mahalanobis distance).

We also analyzed whether the two groups differed significantly in terms of the examined characteristics. In the case of two variables (self-efficacy and authentic leadership), we observed that the mean intensity of these features was statistically significantly higher in the extra LST group than in the no extra LST group. The effect size was similar for both variables. Detailed results are shown in Table 2.

**Table 2:** Comparison of two study groups in terms of variables

Variable	Extra LST group		No extra LST group		t_(df = 327)_^*^	*p*-value*	d** (95%CI)
	M	SD	M	SD			
Care/Empathy	30.02	3.88	29.98	4.11	0.077	0.938	–
Fairness	28.33	3.97	28.86	3.72	−1.197	0.232	–
Self-efficacy	32.35	4.83	30.38	4.28	3.799	0.000	0.44 (0.21; 0.67)
Decision-making skills	11.48	2.76	11.20	2.46	0.935	0.350	–
Authentic leadership	61.98	6.60	59.35	5.99	3.659	0.000	0.42 (0.19; 0.65)

*M* mean, *SD* Standard deviation, *CI* Confidence interval, *Df* degrees of freedom

* Student’s t test

** Cohen’s d coefficient

### Measurement model

Both calculated measures (CMIN = 15.020, df = 8 and CMIN/DF = 1.88) point to the empirical confirmation of the model. CMIN contributions from each group were 7.060 (CMIN/DF = 0.88) for the extra life skills training group and 7.960 (CMIN/DF = 1.00) for the no extra life skills training group. Moreover, based on the test probability values that were obtained (*P* = 0.059) we assumed that the hypothesis about the lack of differences between the theoretical and empirical variance–covariance matrix to be very probable. The measurement of test invariance across groups indicated that the model did not differ statistically in terms of the pathway coefficients between the two study groups (CMIN = 7.404, df = 5, *P* = 0.192).

Since the purpose of testing the model was not only to assess its fit to empirical data from the studied sample but also in relation to the entire population, we calculated the value of the discrepancy function F0 and the value of the RMSEA index adjusted by the number of degrees of freedom. The RMSEA value (0.073, 90% CI (0.001; 0.129), *P* = 0.219) indicated the equality of the two matrices. Additionally, the SRMR value was below the assumed threshold and amounted to 0.046. These results confirmed a good fit of the data to the assumed structural model.

In order to estimate the degree of the fit of the model to the collected data more accurately, fit indexes were determined by comparing the tested model with the independent model, i.e., one in which all of the variables in the model are uncorrelated. The CFI value (0.967) and its adjusted value, i.e., TLI (0.942) were estimated. These results indicate that nearly 100% of the variability of the dependent variable can be explained by the tested model.

### Associations between factors and attitude of authentic leadership

The analysis of the proposed path model showed that all of the relationships were positive. We observed that the following variables have significant direct effects on the output variable of authentic leadership (AL): self-efficacy and care/empathy. For these variables, in the extra LST group, the standardized regression weights amounted to 0.489 and 0.240, respectively. However, in the no extra LST group, the values of these parameters were 0.338 and 0.336, respectively. Regarding the decision-making skills variable, we observed it to significantly influence authentic leadership in the first group (0.236, *P* = 0.002), but this was not the case in the second group (0.120, *P* = 0.065). Regarding the above parameters, the results of test invariance across groups showed no statistically significant differences.

The analysis of the individual path coefficients also showed that the self-efficacy variable had a different impact on the decision-making skills variable depending on its strength. In the first group, the standardized regression weight was only 0.240, whereas in the second group, it was 0.404. On the other hand, the impact of the fairness variable on decision-making skills was similar in both groups (0.269 and 0.277, respectively). Regarding the correlation between fairness and care/empathy, we did not observe any significant differences between the groups (0.687 and 0.728, respectively). A detailed summary of the parameter estimation results of the structural equation model together with the comparative group analysis is presented in Table 3.

**Table 3:** Standardized regression weights and test invariance across groups

Construct	Extra LST group				No extra LST group				CMIN	*p*-value*
	Estimate	SE	CR	*P*-value	Estimate	SE	CR	*P*-value		
SE -- > AL	0.489	0.068	7.214	0.000	0.338	0.060	5.621	0.000	1.957	0.162
C/E -- > AL	0.240	0.073	3.298	0.001	0.336	0.058	5.841	0.000	0.332	0.564
DMS -- > AL	0.236	0.074	3.173	0.002	0.120	0.065	1.848	0.065	1.282	0.258
SE -- > DMS	0.240	0.088	2.724	0.006	0.404	0.054	7.418	0.000	2.543	0.111
F -- > DMS	0.269	0.087	3.078	0.002	0.277	0.057	4.833	0.000	0.000	0.989
F < ->C/E	0.687	0.050	13.806	0.000	0.728	0.032	22.773	0.000	0.076	0.782

*SE* Self-efficacy, *AL* Authentic leadership, *C/E* Care/empathy, *DMS* Decision-making skills, *F* Fairness, *SE* Standard error, *CR* Critical ratio, *CMIN* Chi-square value

* test invariance across groups

Apart from direct effects, we also calculated the value of indirect effects and total effects, for which the influence of the independent variables on the dependent variable is not direct. In this model, indirect effects were present on the fairness ➔ decision-making skills ➔ authentic leadership path (standardized regression weight, in group one and two, respectively: 0.063 and 0.033). Therefore, indirect effects were noticeably weaker in the no extra life skills training group.

**Fig. 3:**
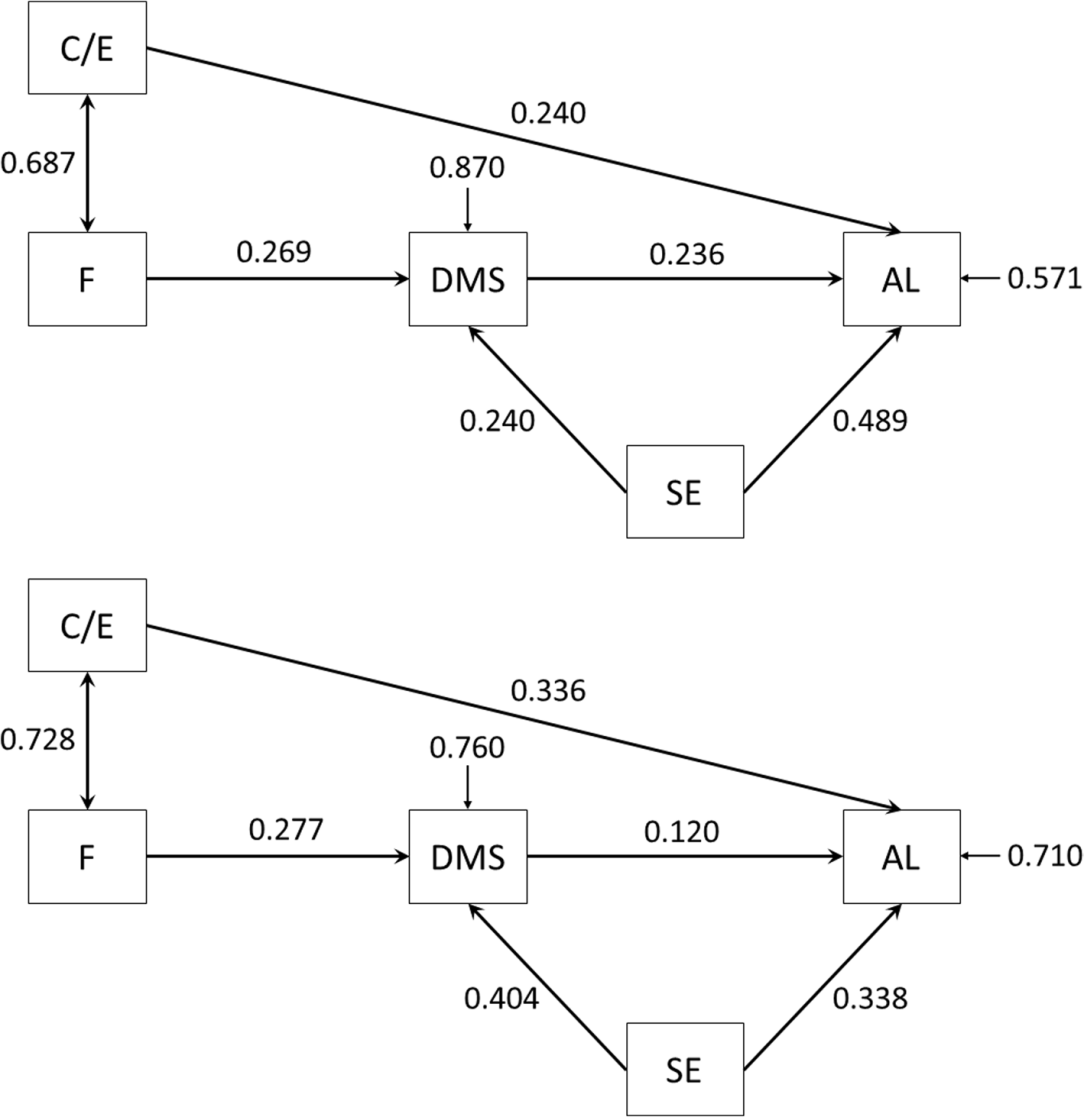
A pathway diagram for the extra LST group (above) and the no extra LST group (below). Correlations between independent variables are indicated with double-sided arrows. Direct effects are indicated with one-sided arrows. The number above the arrow indicates the value of the standardized regression weights. The number next to the arrow shows residual variances. AL—authentic leadership, C/E—care/empathy, DMS—decision-making skills, SE—self-efficacy, F—fairness

It also worth noting that the variable of self-efficacy showed both direct and indirect effects when acting on the dependent variable (authentic leadership) in the extra LST group. In this group, the direct effect value was 0.489, while the indirect effect value was small and amounted to only 0.004. Therefore, in this case, the total effects did not differ much from the direct effects. However, in the no extra LST group, there was no statistically significant indirect effect because no significant relationship was observed between decision-making skills and authentic leadership. Fig. 3 shows a detailed presentation of the parameter estimation results of the structural equation model with all of the direct and indirect effects.

## Discussion

The analyses that were conducted revealed that in public health students, decision-making skills play an important role connecting attitudes (care/empathy), beliefs about fairness regarding group cooperation, and self-efficacy with authentic leadership skills. Thus, the presented results emphasize the complex nature of decision-making skills. They also indicate that these skills are not only important in the context of evidence-based practices for public health [33] and evidence-informed decision making (EIDM) [34], but they also play a key role in developing other competences that are necessary in the work of public health specialists. Therefore, it is reasonable to develop decision-making skills in health students as effectively as possible. Improving these skills increases one’s level of expertise and competence, which, in turn, allows one to take better action to improve public health both on a local as well as global scale. This is in line with ASPHER’s statement [1] as well as literature reports emphasizing that management decision-making is an important factor in evidence-based management in the field of public health. This translates into improving health care management practices through the use of the evidence-based approaches to ensure the quality of managerial decisions [35]. Moreover, adequate decision-making skills are extremely useful for distinguishing fact from myth/opinion, and there is a plethora of the latter in the field of public health [36]. Additionally, Moodie [37] considers vision and decision making to be key leadership skills in the field of public health.

The study demonstrated that decision-making skills have a positive impact on developing authentic leadership skills. The crucial role of these skills in public health is underlined by, e.g., Stander [38], who emphasizes that the implementation of authentic leadership by healthcare managers may lead to greater optimism, trust in the organization, and work engagement. Similar conclusions were drawn by Coxen [39], who points to the benefits of encouraging employees to become authentic leaders. In addition, researchers dealing with the topic in question recommend that all healthcare institutions invest in the continuous evaluation of the level of leadership competences of the managerial staff as well as in authentic leadership training [40, 41]. However, it is worth highlighting that leadership skills training should begin at university. The available literature indicates, however, that despite the fact that for many years, the importance of leadership competences in public health has been growing and has been widely discussed, there is a lack of leadership training at universities [42, 43]. Czabanowska, Lachance, and Konings emphasize the need for public health education programs in Europe to introduce courses that develop and improve leadership competences of future public health specialists [44–46].

In this study, was observed a positive correlation between an empathic attitude (care/empathy) and authentic leadership skills, which is consistent with the findings of other researchers who emphasise that empathy is an important component of leadership skills [6, 7]. It should be noted that the empathic attitude associated with openness to one’s own and other people’s emotions makes up key components of authentic leadership such as relational transparency (i.e., being open about one’s own ideas and emotions) and an internalized moral perspective (i.e., moral integrity) [8]. Interestingly, in these study, an empathetic attitude had no direct impact on decision-making skills, which may suggest the complex role of empathy in making various health-related decisions and there is thus a lack of the universality in this parameter across health-related professions. Such an opinion was expressed in an integrative literature review that included twenty-three papers. Nonetheless, it should be remembered that this study involved a variety of healthcare professionals (nurses, physicians, occupational therapists, physiotherapists, mixed clinician samples, and unspecified infectious disease experts), but no public health specialists [47]. Therefore, research on the role of emotions (emotional intelligence) in decisions made by public health leaders is necessary, and the obtained results require further supporting evidence.

The conducted analyses also point to the important role of self-efficacy in developing decision-making skills and leadership skills. There are many reports suggesting a direct relationship between self-efficacy and leadership skills [7, 8] as an important factor in self-development [25]. It should be emphasized that self-efficacy increases self-awareness (awareness of one’s strengths and weaknesses) as well as encourages critical reflection on one’s own skills. It is an inner belief that a person may not only achieve a set goal, but motivation for self-improvement is internal as well [15], and this is consistent with the concept of authentic leadership [7, 8] and reports by other researchers [48].

The analysis did not reveal any differences regarding the developed structural model of factors related to individual dispositions (fairness, care/empathy, self-efficacy) on the development of decision-making skills and authentic leadership skills. The declared participation in the extra life skills training group was not a differentiating variable. The obtained results should be interpreted with caution because participation in the extra LST group was declarative and subjective. We did not verify the type of extra LST received. As far as collecting data from eight Polish universities is concerned, obtaining more detailed qualitative data was difficult for technical reasons. The extra LST offered at these universities is diverse, and a quantitative analysis is impossible for legal reasons. The developed model should be verified in further studies against more detailed information about LST. It should be noted, however, that these were additional LST courses, and the training offered in the curriculum was not taken into account. However, it should not be assumed that the lack of differences in the analyzed groups (extra LST group vs. no extra LST group) suggests a low effectiveness of extra LST. This would be too much of a simplification and an over-interpretation. It is worth noting that one’s attitude is relatively permanent—although it may undergo modifications, similarly to self-efficacy. Change takes time and effort. Therefore, further analyses should include extra LST focused on self-improvement and reflection on one’s own strengths and weaknesses. This would be consistent with the suggestions of other researchers [45, 48].

There is a lot of evidence in the available literature that indicates that leadership training is highly effective when conducted among students. Participants of leadership skills improvement programs are characterized as having a higher level of leadership skills compared to students who have not undergone leadership training. Moreover, thanks to participating in such training, they build additional soft skills, such as communication skills, decision-making skills, or teamwork [49–52].

The effectiveness of leadership training and the level of authentic leadership skills of a particular person are influenced by many factors, both individual and institutional, legal and environmental. However, the theoretical model developed in this study concerns the academic community, not the professional one, and may constitute the basis for creating a universal leadership training in Poland, taking into account individual conditions.

### Practical implications

Analyzing the results, it can be observed that the development of leadership skills can take place directly, although this is a very complicated process, or indirectly, by strengthening one’s self-efficacy, empathic attitude, and the belief that individuals in a group should make a fair contribution to that group. Therefore, it seems justified to design appropriate courses/training that would allow public health students to acquire these skills. Having analyzed the developed structural model, we can assume that the first step in developing leadership skills in public health students should involve improving self-efficacy, namely identifying one’s strengths and weaknesses combined with self-reflection and persistence in pursuing one’s goals. This is all the more important in light of the available sources emphasizing that self-awareness and confidence are factors contributing to increased exemplary leadership practices as well as to an improved leadership development framework [45, 53]. The essential role of integrity as a competence in public health leaders is also emphasized by Bayer et al. [48]. Activities aimed at developing moral and empathic attitudes should be undertaken at the same time as those increasing self-awareness and reflection, as they allow for the creation of a friendly work setting and relate to skills such as teamwork, relationship building, organizational awareness, and project management [45]. This is crucial also with respect to competences such as effective communication skills, networking skills, cultural competency skills, or negotiation skills, among others [48]. Later on, training aimed at strengthening decision-making skills should be introduced, as they are the underpinnings of leadership skills development. This way, the multi-dimensional development of leadership skills among public health students can be achieved.

### Limitations

Our study has some limitations. It must be emphasized that the purpose of our study was not to test the effectiveness of leadership training.

First, the declarative measurement of participation in extra LST, which could have contributed to no differences being noted between the analyzed groups, is one of important limitations of the presented study. Additionally, we do not have precise information on what kind of training the students from extra LST group took, how long it lasted, what form it was and what exactly was the scope of the topic. A second limitation is the empirical validation of the theoretical model based on the data from cross-sectional studies. Therefore, the obtained results should be treated with caution and assumptions should not be made about changes in trends over time. Third, we did not collect data on the number of respondents who refused to participate in the study. The size of this group and their sociodemographic status are not known. Therefore, it should be assumed that individuals who felt insecure about their leadership skills may have refused to participate in the study. Additionally, we did not analyze the intensity of social approval or the tendency to respond in-line with the researcher’s expectations. This is especially important as far as research on attitude is concerned. In the end it should be notice that in this study we took into account the influence of life skills on the level of authentic leadership skills among students at the beginning of their professional journey. Therefore, the impact of other factors that ma affect the level of authentic leadership competences are unknow.

## Conclusions

The development of authentic leadership skills in public health students draws on the appropriate development of individual dispositions (e.g., self-efficacy, fairness, empathetic attitudes, and DMS). Therefore, a verified theoretical model showed that course aimed at strengthening these competences should be modular, should focus on self-improvement and critical reflection, and should be spread over time to enable and encourage each participant to grow and flourish at their own pace.

## Data Availability

All data generated or analysed during this study are included in this published article [and its supplementary information files].
